# Canonical and non-canonical roles for ATG8 proteins in autophagy and beyond

**DOI:** 10.3389/fmolb.2022.1074701

**Published:** 2022-12-19

**Authors:** Steven Edward Reid, Srinivasa Prasad Kolapalli, Thorbjørn M. Nielsen, Lisa B. Frankel

**Affiliations:** ^1^ Danish Cancer Society Research Center, Copenhagen, Denmark; ^2^ Biotech Research and Innovation Center, University of Copenhagen, Copenhagen, Denmark

**Keywords:** autophagy, lipidation, Atg8, post-translational modification (PTM), secretory autophagy, single membrane

## Abstract

During autophagy, the ATG8 family proteins have several well-characterized roles in facilitating early, mid, and late steps of autophagy, including autophagosome expansion, cargo recruitment and autophagosome-lysosome fusion. Their discovery has importantly allowed for precise experimental monitoring of the pathway, bringing about a huge expansion of research in the field over the last decades. In this review, we discuss both canonical and non-canonical roles of the autophagic lipidation machinery, with particular focus on the ATG8 proteins, their post-translational modifications and their increasingly uncovered alternative roles mediated through their anchoring at different membranes. These include endosomes, macropinosomes, phagosomes and the plasma membrane, to which ATG8 proteins can bind through canonical or alternative lipidation. Beyond new ATG8 binding partners and cargo types, we also explore several open questions related to alternative outcomes of autophagic machinery engagement beyond degradation. These include their roles in plasma membrane repair and secretion of selected substrates as well as the physiological implications hereof in health and disease.

## Introduction

Eukaryotic cells are continuously exposed to external stressors from the environment throughout their lifetime. This demands a highly controlled adaptive cellular response, in which cells undergo rapid changes to cope with harsh conditions. By adapting their metabolism and protecting themselves against potential damage, cells can maintain homeostasis and function, also in the presence of environmental challenges. However, if not managed carefully and correctly, the consequences can be detrimental. Autophagy is one such adaptive cellular process, which functions at basal levels and is highly induced in response to various types of stress. These include nutrient deprivation, growth factor withdrawal, reactive oxygen species, DNA damage, hypoxia, infection, and various other cytotoxic insults (Dikic and Elazar, 2018). By degrading damaged cellular organelles, bulk cytoplasm, protein aggregates and invading pathogens, autophagy is mainly regarded as a cytoprotective mechanism, yet high levels of autophagy can in certain settings cause cell death (Liu and Levine, 2015).

**FIGURE 1:**
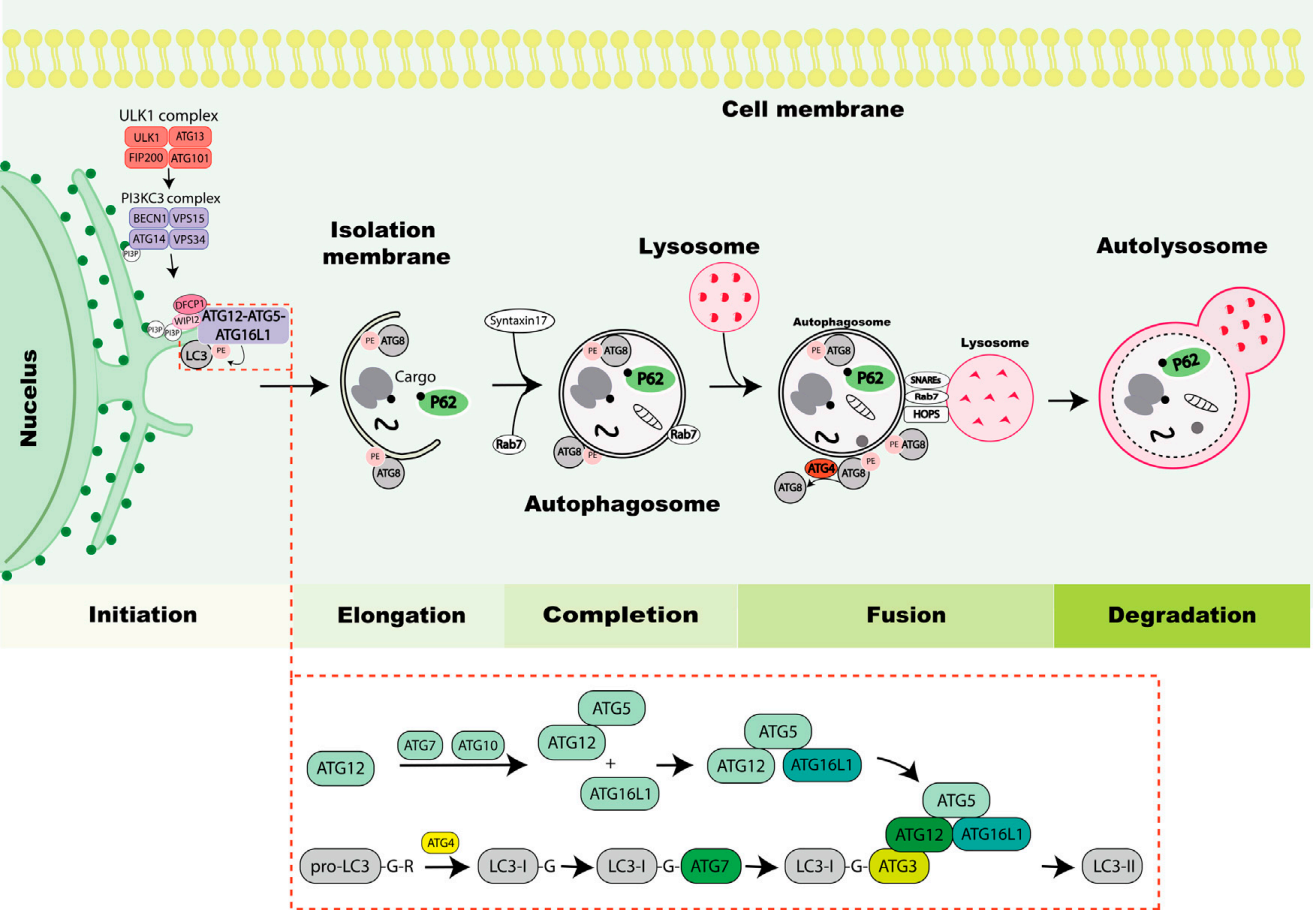
Overview of the autophagy process. The autophagy process is initiated by activation of the Unc-51-like kinase 1 (ULK1) complex consisting of ULK1, autophagy-related protein 13 (ATG13), RB1-inducible coiled-coil protein 1 (FIP200) and ATG101. The ULK1 complex induces nucleation of the phagophore by phosphorylating components of the PI3KC3 complex consisting of Beclin 1, vacuolar protein sorting 34 (VPS34), vacuolar protein sorting 15 (VPS15) and ATG14. The PI3KC3 complex mediates the production of phosphatidylinositol-3-phosphate (PI3P) at the ER to mediate the formation of the omegasome. Then, zinc-finger FYVE domain-containing protein 1 (DFCP1) and WD repeat domain phosphoinositide-interacting protein 2 (WIPI2) containing PI3P-interacting regions are recruited to the omegasome, which are required for the recruitment of lipidation machinery consisting of ATG12-ATG5-ATG16L1 by binding to ATG16L1. The stepwise process of ATG8 lipidation is depicted in the lower box, exemplified with LC3 and resulting in LC3 conjugation to phosphatidylethanolamine (PE) (depicted in figure as LC3-II) which drives elongation of phagophore. Cargos including protein aggregates, organelles or ribosomes are typically marked by ubiquitin eat-me signal (black dot) leading to their sequestration by autophagy receptors such as P62 that mediates their recruitment to the phagophore. The ATG8 proteins mediate closure of the autophagosome by recruiting Syntaxin17. Further, Rab7 is recruited to autophagosomes and facilitates the fusion process. During the fusion process ATG4 de-lipidates ATG8 on the outer membrane of the autophagosome and the inner membrane is degraded within the lysosome. The process of autophagosome and lysosome fusion is mediated by SNAREs, HOPS and small GTPases (Rab7). Finally lysosomal hydrolases degrade the autophagic cargos.

During autophagy, intracellular cargos are sequestered by a growing double membrane, ultimately sealing to form the autophagosome and subsequently transported to lysosomes for their degradation. Two key upstream signaling nodes which function to transmit the cellular stress signals to the autophagy machinery include the mammalian target of rapamycin (mTOR) and AMP-activated protein kinase (AMPK). Landmark genetic screens in yeast have led to the discovery of several evolutionarily conserved autophagy related (ATG) proteins with conserved orthologs in higher eukaryotes (Klionsky et al., 2003), opening in depth understanding of how the process is regulated. Briefly, autophagy can be classified into five main steps of initiation, elongation, completion, fusion and degradation (Figure 1). Key machinery orchestrating these steps includes i) the ATG1/Unc-51 like kinase (ULK1) complex that triggers the autophagy cascade, ii) the phosphatidylinositol 3-phosphate kinase catalytic subunit type 3 (PI3KC3) complex activated by ULK1 and involved in vesicle nucleation by recruiting phosphatidylinositol 3-phosphate, iii) two ubiquitin like conjugation systems (Atg12 and Atg8) promoting vesicle expansion by lipidating ATG8s, which are also involved in cargo recruitment and autophagosome closure (Fujita et al., 2008; Weidberg et al., 2010) and iv) SNAP Receptors (SNAREs), homotypic fusion and protein sorting (HOPS)–tethering complex and small GTPase recruitment to autophagosomes by ATG8s to mediate autophagosome-lysosome fusion (Manil-Segalen et al., 2014; Gao et al., 2018; Gu et al., 2019). After fusion, the contents present in the autolysosomal lumen are degraded by lysosomal hydrolases, whereas autophagosomal components on the autolysosomal membrane are recycled by the process recently coined autophagosomal components recycling (ACR) (Zhou et al., 2022).

## ATG8 lipidation

ATG8 is a ubiquitin-like protein originally identified in *S. cerevisiae* (Ichimura et al., 2000; Ohsumi, 2001). A subsequent sequence analysis identified mammalian microtubule associated protein 1 light chain 3 (LC3) and GABA type A receptor-associated protein (GABARAP) proteins to have homology with ATG8, demonstrating their evolutionary conservation. A total of seven functional ATG8 genes were identified in humans (LC3A, LC3B, LC3B2, LC3C, GABARAP, GABARAPL1, GABARAPL2), of which LC3B2 express at a low level (Legesse-Miller et al., 1998; Wang et al., 1999; Kabeya et al., 2000). The lipidation of ATG8 occurs in a manner that is analogous to the conjugation of ubiquitin to target proteins. Newly synthesized pro-LC3s/GABARAPs are primed by ATG4 where the C-terminal extension of one or more amino acid residues is proteolytically cleaved. This exposes a glycine residue that later becomes conjugated to phosphatidylethanolamine (PE) by a series of enzymatic reactions involving E1-, E2- and E3-like complexes (Ichimura et al., 2000; Kirisako et al., 2000) (Figure 1). After priming by ATG4, ATG8s are transferred to the E1-like enzyme ATG7, and the covalent linkage of PE to a specific glycine residue of ATG8 is performed by collaborative activities of the E2-like ATG3 and E3-like ATG12-ATG5-ATG16L1 complexes (Hanada et al., 2007; Geng and Klionsky, 2008; Sakoh-Nakatogawa et al., 2013). *In vitro* experiments showed that ATG8 lipidation can occur without E3-like enzymes, but their presence accelerates the reaction (Martens and Fracchiolla, 2020). The PE conjugated ATG8s are also referred to as lipidated ATG8s and this process is crucially important for their recruitment to the membrane where ATG8s serve as platform for recognition by autophagy receptors, adaptors, and other autophagy proteins (Behrends et al., 2010; Wild et al., 2014; Johansen and Lamark, 2020). Most cargos do not bind to ATG8s directly but are usually sequestered by receptors which can bind to ATG8s by a short linear motif called ATG8-family interacting motif (AIM) or LC3-interacting region (LIR) (Birgisdottir et al., 2013; Popelka and Klionsky, 2015). The attachment of PE to ATG8s is reversible and ATG8-PE is present on both the inner and outer phagophore. However, the ATG8s are not present on the outside of fully formed autophagosomes, as ATG8-PE present on the outer surface of the autophagosome is deconjugated and released by a second ATG4 dependent cleavage (Maruyama and Noda, 2017). ATG8 lipidation is considered a hallmark of autophagy and is used as a marker to monitor autophagy flux experimentally (Nakatogawa et al., 2007). ATG8 de-lipidation is inherently slow, but functionally important and regulated by all ATG4 homologs (Kirisako et al., 2000; Nair et al., 2012; Kauffman et al., 2018). Functional discrepancies between the individual mammalian ATG8 proteins are currently not fully understood. Knockout of all family members showed that the ATG8s are dispensable for autophagosome formation, but that their absence resulted in smaller autophagosomes and the slowing of pathway kinetics (Nguyen et al., 2016). Rescue experiments suggested the particular importance of GABARAP subfamily members for the later pathway stages of maturation and autophagosome-lysosome fusion, where they also act as platforms for recruitment of mediators of fusion (Weidberg et al., 2010; McEwan et al., 2015; Wang et al., 2015; Kumar et al., 2018; Gu et al., 2019). GABARAP has also been linked to lysosome biogenesis and autophagy *via* activation of transcription factor EB (TFEB), a key transcription factor that promotes autophagosome formation and their fusion with lysosomes (Goodwin et al., 2021). Beyond canonical lipidation, it was recently shown that all ATG8s can undergo alternative conjugation to phosphatidylserine (PS), instead of PE, during LC3-associated phagocytosis (LAP) or influenza A infection (Durgan et al., 2021). However, it may play a broader physiological role during other types of non-canonical autophagy. Since this alternative conjugation is not detected during canonical autophagy, it may constitute a specific molecular signature that triggers alternative signaling pathways, however, so far, the functional roles of this alternative lipidation remain poorly understood.

## Post translational modifications of ATG8s

Beyond lipidation, several post-translational modifications (PTMs) are known to occur directly on ATG8 proteins, serving as an important regulatory axis of autophagy. These include phosphorylation, ubiquitination and acetylation, and are increasingly viewed as fine tuners of the pathway. They can ensure a correct sequential order of events, orchestrate directionality of vesicle transport, and impact key ATG8 features such as lipidation status, interaction partners and regulation of cargo degradation. Among the first studies showing a role for phosphorylation of LC3 was Cherra et al., revealing that protein kinase A (PKA) phosphorylates rat LC3 at Serine 12 leading to its reduced recruitment to the autophagosome and negatively impacting autophagy (Cherra et al., 2010). More recently it was shown that LC3C and GABARAP-L2 are phosphorylated on the surface-exposed serines S93, S96 and S87, S88, respectively, by TANK-binding kinase 1 (TBK1), leading to destabilization of the ATG8-ATG4B complex. It was suggested that this mechanism serves to protect against premature de-lipidation of ATG8s on newly forming autophagosomes, hence preserving correct unidirectional autophagy (Herhaus et al., 2020). Furthermore, LC3B can be phosphorylated by serine/threonine kinase 4 (STK4) at Thr50, which impairs LC3B binding to FYVE and coiled-coil domain autophagy adapter 1 (FYCO1) and thereby reduces directional transport of autophagosomes to the cell periphery (Nieto-Torres et al., 2021). Interestingly, the same site on LC3B is phosphorylated by additional kinases including never in mitosis A (NIMA)-related kinase 9 (NEK9), which inhibits selective degradation of p62 and neighbor of BRCA1 gene 1 Protein (NBR1) (Shrestha et al., 2020). Although the precise mechanism is not fully understood, the authors showed that a LC3B T50E phospho-mimicking mutant also inhibited interactions with ATG4B, ATG7 and syntaxin-17 (STX17). Interestingly, the Thr50 site is conserved among the LC3 family members, but is not present in the GABARAP proteins (Shrestha et al., 2020). The functional consequences of ATG8 modifications remain unclear, but are likely to cause alterations in relation to protein folding, structure, stability or binding partners. Indeed, NMR spectroscopy has been used to determine the three-dimensional structure of LC3C, revealing that phosphorylation *in vitro* by PKA on LC3C (Ser18) caused a conformational change in the protein that affected its LIR-binding interface (Krichel et al., 2019). Such studies help to shed light on how PTMs can modulate key ATG8 interactions. Acetylation is another PTM known to occur on ATG8s, and it was shown that a carefully coordinated cycle of acetylation and de-acetylation impacts the nuclear-to-cytoplasmic shuttling of LC3. This importantly enables LC3’s effective redistribution to the cytoplasm and its association to Atg7 during starvation-induced autophagy, which is dependent on Sirt1-induced de-acetylation of K49 and K51 (Huang et al., 2015a). Another study showed that loss of the methyltransferase Opi3 leads to abnormally lipidated LC3B due to an accumulation of phosphatidylmonomethylethanolamine (PMME) and consequently, conjugation of ATG8 to PMME (Sakakibara et al., 2015). Beyond irregular localization patterns of ATG8, it was further shown that ATG8 cannot be fully deconjugated from PMME by ATG4, ultimately leading to deficient ATG8 recycling. Finally, ubiquitination of ATG8s is also known to impact autophagy. The E2/E3 ligase baculoviral IAP repeat-containing protein 6 (BIRC6) along with E1 enzyme ubiquitin-like modifier-activating enzyme 6 (UBA6) promote LC3B monoubiquitination leading to proteasomal degradation and inhibition of autophagy (Jia and Bonifacino, 2020). Counteracting this, LC3B ubiquitination is reversed by the action of the deubiquitinating enzyme ubiquitin carboxyl-terminal hydrolase 10 (USP10), which increases LC3B levels and autophagy activity, in turn impacting the clearance of autophagy receptors p62 and NBR1, as well as puromycin-induced aggregates (Jia and Bonifacino, 2021). As ATG8 family members can localize to several subcellular regions, this opens the possibility for coordinately regulated spatiotemporal effects. For instance, the centriolar satellite MIB E3 ubiquitin protein ligase 1 (MIB1) was found to promote K48-linked ubiquitination of GABARAP on N-terminal residues not present on LC3 family members, specifically regulating the centrosomal pool of GABARAPs (Joachim et al., 2017). It is clear that PTMs on ATG8s can fine tune the autophagy pathway in various ways, suggesting that these may act as molecular switches that quickly allow cells to modulate or reprogram the autophagic response when facing different types of cellular stress.

## Recruitment of ATG8 to single membranes

**FIGURE 2:**
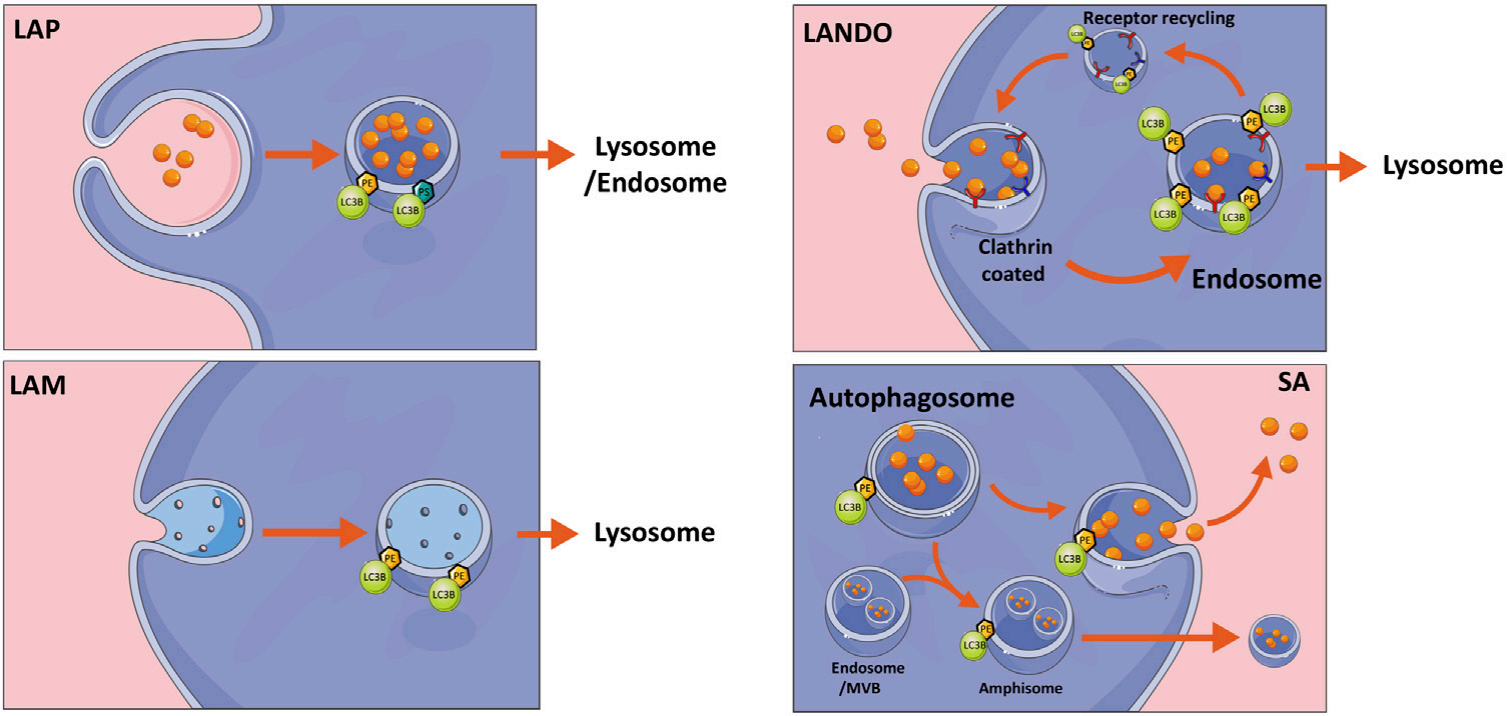
Non-canonical autophagy pathways. Overview of non-canonical autophagy pathways including LC3-associated phagocytosis (LAP), LC3-associated micropinocytosis (LAM), LC3-associated endocytosis (LANDO), all of which are classed as conjugation of ATG8 to single membrane (CASM) mechanisms and finally secretory autophagy (SA). Briefly LAP and LANDO internalize extracellular material for either degradation or endosomal recycling, whereas LAM internalizes damaged membrane for removal. SA can release autophagosome contents to the extracellular space either directly as free molecules or within multivesicular bodies (MVB). The latter requires fusion with endosomes to create an amphisome intermediate. See text for further description. The figure was created by modifying illustrations from servier medical art licenced under a Creative Commons Attribution 3.0 Unported License: (http://creativecommons.org/licenses/by/3.0/).

In addition to their classical conjugation to double membranes, ATG8s can through non-canonical pathways be recruited to single membranes including endosomes, phagosomes, macropinosomes and plasma membrane (Yang et al., 2012; Fletcher et al., 2018; Sonder et al., 2021). Depending on the membrane type, this can have alternative outcomes spanning from degradation to secretion or membrane repair (Kakanj et al., 2022; Vats and Galli, 2022). The process, which has also been referred to as conjugation of ATG8 to single membranes (CASM) is summarized in Figure 2, can both involve classical ATG8 conjugation to PE, or alternatively to PS, as described above (Durgan et al., 2021; Hooper et al., 2022). Interestingly, single membrane lipidation has limited dependency on the classical upstream autophagy initiation machinery including mTOR and ULK1 complexes, while still engaging most of the canonical ubiquitin-like conjugation program.

LAP is a process utilizing single membrane ATG8 lipidation, resulting in the decoration of phagocytic vesicles with LC3, which are destined for degradation by the autophagy pathway. LAP can be regarded as a hybrid system between autophagy and phagocytosis, both of which constitute important and related arms of the host’s first line of defense against invading microbes (Yang et al., 2012). While autophagy sequesters and degrades intracellular material, phagocytosis engulfs external particles. Yet common to both pathways is the eventual degradation of cargos through delivery to lysosomes. While LAP is not activated by nutrient stress, it is rather activated by phagocytosis-inducing external stressors including a variety of ligands, immune complexes, pathogens or signals including metabolites from cells undergoing apoptosis (Martinez et al., 2011; Henault et al., 2012; Kyrmizi et al., 2013; Heckmann and Green, 2019). Although our understanding of the mechanism of LAP formation has improved, it is still not clear how the ligation of receptors stimulates the assembly of autophagy components at the phagosome. However, it is known that the recognition of foreign particles is followed by phagosome formation and recruitment of the PI3KC3 complex. While the canonical autophagy-regulating PI3KC3 complex consists of autophagy and beclin1 regulator 1 (AMBRA1) and ATG14L, the LAP-related PI3KC3 complex does not contain AMBRA1 or ATG14L, but instead run domain Beclin-1-interacting and cysteine-rich domain-containing protein (RUBCN) and UV radiation resistance-associated gene protein (UVRAG) (Sanjuan et al., 2007). Unlike conventional autophagy, LC3-conjugation is not required for membrane closure in LAP but is required for LAP fusion with the lysosome or endosome (Henault et al., 2012; Martinez et al., 2015). Importantly, loss of functional lipidation machinery leads to failure of LAP that is associated with inflammation, autoimmune disorders, trafficking, secretion, and vision impairment (Kim et al., 2013).

A mechanistically distinct process called LC3-associated endocytosis (LANDO) has drawn recent attention in the field of neurodegeneration, due to its ability to promote clearance of amyloid-beta (AB) protein aggregates in microglial cells and inhibition of neuronal inflammation. In addition, LANDO promotes the recycling of receptors required for efficient clearance of AB. Using a murine model of Alzheimer’s Disease, the authors showed that absence of LANDO led to accelerated neurodegeneration (Heckmann et al., 2019). Similar to LAP, LANDO does not require upstream autophagy regulators. However, recent molecular dissections of the ATG16L1 protein, which is known to direct ATG8 to sites of lipidation, revealed that its WD40 domain is essential for mediating LC3 lipidation at single membranes, while being dispensable for canonical autophagy (Fletcher et al., 2018).

A unique form of macropinocytosis involving parts of the autophagy machinery, termed LC3-associated micropinocytosis (LAM), also involves LC3 lipidation at single membranes (Sonder et al., 2021). Using a model of laser-induced injury of the plasma membrane, ATG7-dependent formation of LC3-positive vesicles was observed around the plasma membrane repair area, serving to remove damaged material from the plasma membrane and restore membrane integrity. LAM is triggered just minutes after initial membrane resealing, allowing internalization of damaged membrane to restore normal plasma membrane composition. Internalized LC3 positive vesicles eventually fused with lysosomes and as with other types of CASM, this was shown to be an ULK1-, ATG13-, and WD repeat domain phosphoinositide-interacting protein 2 (WIPI2)-independent non-canonical autophagy process. Similar to LAP, RUBCN was implicated in the formation of LC3-positive vesicles, but was dispensable for their repair capacity and for the ability to internalize damaged membrane (Sonder et al., 2021). In a related process, Kakanj et al. (Kakanj et al., 2022) recently showed a non-canonical role of the autophagy machinery in wound healing, whereby cells at the wound edge can fuse and form multinucleated cells. Wounding induces autophagy and is required for efficient wound repair from plants to animals. Using *Drosophila* as a model, it was shown that the lateral plasma membrane between two epithelial cells can be removed, whilst leaving the apical and basal membranes intact, to result in cellular fusion and a multinucleated cell. This process required autophagosome initiation and expansion but not lysosomal fusion (Kakanj et al., 2022). When autophagy was induced, this process was also observed to occur in the unwounded cells without affecting epithelial barrier function (Kakanj et al., 2022). These studies shed light on a rather new concept of specifically employing the autophagy machinery to remove membrane material, in order to rapidly restore or maintain barrier integrity at the cellular or multicellular level.

Monitoring ATG8 (and particularly LC3B) puncta has for many years constituted one of the most widely used cellular assays to estimate autophagy levels (Klionsky et al., 2021). Yet as discussed above, considering the expanding repertoire of membranes that ATG8 proteins conjugate to, it is worthy to note that caution should be taken in the interpretation data arising from ATG8 puncta quantifications. Indeed, these puncta may represent multiple other intracellular structures and only through careful co-localization studies with additional markers, complemented by methods such as electron microscopy and biochemical assays, can one be fully conclusive about the nature and function of such puncta.

## Secretory autophagy, its molecular triggers and implications in cancer

**FIGURE 3:**
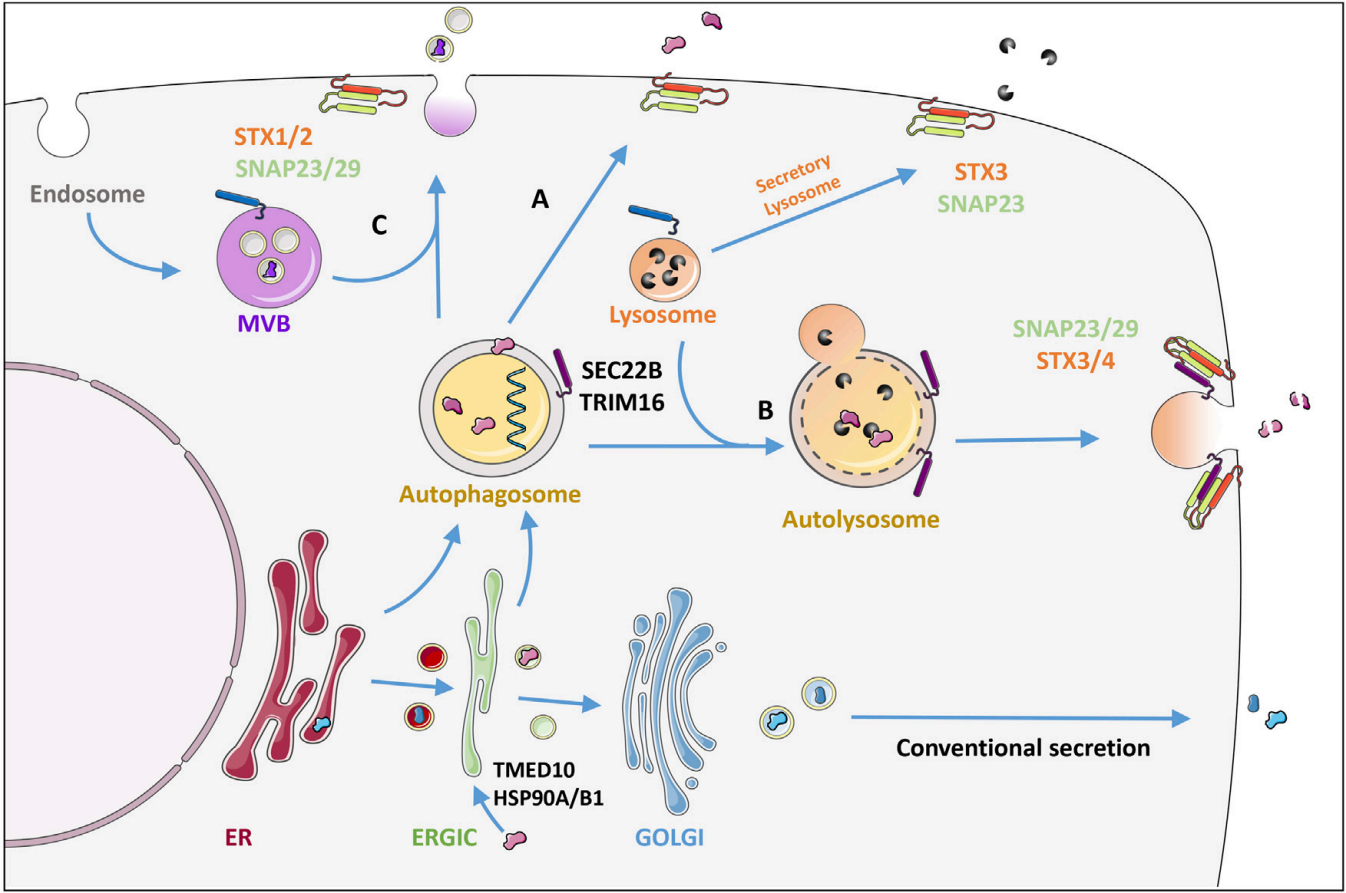
Secretory autophagy: an unconventional secretory pathway. In contrast to conventional secretion where proteins are trafficked through the ER-Golgi, proteins can also enter the autophagosome through the ER or directly from the ERGIC together with the membrane that forms the autophagosome. TMED10 together with the chaperones HSP90A and HSP90B1 have been shown to allow protein cargo to enter the ERGIC post-translationally. TRIM16 recruits IL1B directly to the LC3 labelled membranes for secretion. Autophagosome cargo can either be directly released by fusion with the plasma membrane **(A)** or be released following lysosomal fusion where R-SNARE Sec22 on the autophagosome/autolysosome membrane delivers the vesicle specifically to the plasma membrane where it interacts with syntaxins (STX) 3 or 4 together with the SNAPs 23 and 29 **(B)**. Alternatively fusion of the autophagosome with multivesicular bodies (MVB) **(C)** releases cargo within extracellular vesicles by binding to STX 1 or 2 also with SNAPs 23/29. This is in contrast to secretory lysosomes which release the digestive enzymes of lysosomes using STX3 and SNAP 23. The mechanism of release determines whether the cargo is released intact **(A)**, digested **(B)** or within a protective vesicle **(C)**. See text for more details. Figure was created by modifying illustrations from servier medical art licenced under a Creative Commons Attribution 3.0 Unported License: (http://creativecommons.org/licenses/by/3.0/).

While typical secretory proteins contain an N-terminal leader peptide for efficient trafficking through the ER and Golgi before release into the extracellular space (Vats and Galli, 2022), proteins that lack a signal peptide are released by one of several unconventional secretion mechanisms (Vats and Galli, 2022). The most well-known is *via* exosomes that are derived from late endosomal membranes and packaged in multivesicular bodies before release. Alternatively, the autophagic machinery is increasingly recognized for its implications in cellular secretion. Although the term secretory autophagy (SA) has not been explicitly defined, it essentially includes any unconventional secretion involving the canonical autophagy machinery components (New and Thomas, 2019). During SA, the cargo sequestered into the phagophore is packaged into autophagosomes and targeted towards the plasma membrane instead of being directed to lysosomes for degradation Figure 2 (Vats and Galli, 2022). Upon plasma membrane delivery, the vesicle contents are secreted extracellularly either as soluble factors, membrane bound vesicles or within multivesicular bodies (Figure 3) (Jeppesen et al., 2019). The intact extracellular vesicles secreted *via* secretory autophagy are typically small sized extracellular vesicles containing LC3B, making them distinguishable from CD63 and ESCRT-I complex subunit (TSG101) positive exosomes (Chen et al., 2019). In a related process, autophagosomes that have already fused with the lysosomes and degraded their contents can become ‘secretory lysosomes’ that release their degradation products extracellularly (DeSelm et al., 2011; Ushio et al., 2011; New and Thomas, 2019). Proteins known to be secreted *via* SA include transforming growth factor beta-1 proprotein (TGFB1), high mobility group protein B1 (HMGB1), interleukins (IL)-1B, 6, 8, 18, matrix metalloproteinase-9 (MMP9) and ferritin (Kimura et al., 2017; New and Thomas, 2019; Martinelli et al., 2021). Studies focused on these secreted factors have led to a gained appreciation of the pathway’s importance in normal physiology including the inflammatory response as well as in a wide array of diseases (Cadwell et al., 2008; DeSelm et al., 2011; Torisu et al., 2013; Bel et al., 2017). Hence, defects in secretory autophagy have been linked to numerous pathologies including Crohn’s disease, asthma, type-II diabetes, Alzheimer’s Disease, Parkinson’s Disease, several cancer types as well as bacterial and viral infections (New and Thomas, 2019).

A central question lies in how the leaderless protein cargo are selectively taken up by SA. Moreover, the molecular switches triggering secretion rather than degradation remain unclear. Below we explore some recently elucidated mechanistic insight, suggesting that these outcomes have a high dependency on the initial triggers and cell types.

Studies focusing on the SA substrate IL1B have revealed interesting mechanisms for its selective uptake during SA. For instance, it has been shown that IL1B can be imported post-translationally into the ER-Golgi intermediate compartment (ERGIC), which is a membrane source for autophagosomes (Zhang et al., 2020). This involves the transmembrane spanning protein transmembrane emp24 domain-containing protein 10 (TMED10), which forms oligomers to generate a membrane pore. TMED10 directly binds to a motif located in the cargo *via* its C-terminal domain. The protein cargos are first required to be unfolded to allow them to be fed through the pore, which is done *via* the help of chaperones heat shock protein HSP90A and HSP90B1 (Zhang et al., 2020). The process of unfolding may expose the binding motif to TMED10, yet Zhang et al. also revealed that the cargo motif can enhance TMED10 self-association. This selection mechanism of leaderless protein cargo was confirmed for several other of the IL1 family members as well as cargo such as Tau and annexin A1, but not all known leaderless proteins including HMGB1 or alpha-synuclein (Zhang et al., 2020). Interestingly TMED10 limits autophagy by modulating mTOR activity and by directly binding to and inhibiting ATG4B (Shin and Cho, 2019; Shin et al., 2019). An independent study showed that in response to lysosomal damage, tripartite motif-containing protein 16 (TRIM16) together with Galectin-8 can promote IL1B secretion *via* SA. TRIM16 directly binds to IL1B and recruits it to LC3B positive membranes for secretion. Specificity of vesicle trafficking involves dedicated SNAREs in order to successfully deliver IL1B to the plasma membrane. TRIM16 also binds the autophagosome R-SNARE; SEC22B. SEC22B then delivers the vesicle specifically to the plasma membrane by interacting with syntaxins (STX) 3 or 4 together with the synaptosomal-associated proteins (SNAPs) 23 and 29 allowing fusion and release of vesicle contents. This was not affected by the autophagosome-lysosome R-SNARE: STX-17 (Kimura et al., 2017).

Additionally investigated SA substrates include MMP9 and TGFB1. SA of MMP9 requires the stress responsive chaperone FK506-binding protein 51 (FKBP51) helping to bridge SEC22B and the plasma membrane Q-SNARE enabling vesicle-membrane fusion (Martinelli et al., 2021). SA of MMP9 is increased in response to glucocorticoid-induced stress where it cleaves extracellular brain derived neurotrophic factor (BDNF) precursor (Martinelli et al., 2021). For latent TGFB1 secretion, Rab8B is responsible for both autophagosome maturation and fusion with the lysosome. Whereas Rab8A, which is required for polarized sorting to the plasma membrane, was shown to be responsible for targeting TGFB1-containing autophagosomes to release their contents (Nuchel et al., 2018).

The mechanisms that guide the switch from degradation to selective secretion are currently unclear. It is likely that the balance between both pathway arms can be used to fine tune inflammatory signaling pathways and allow for a rapid and appropriate cellular response, by adjusting degradation and secretion of key inflammatory mediators. Studies on Golgi reassembly-stacking protein 2 (GORASP2), which aids the fusion of autophagosomes and lysosomes provide us with some understanding of how the switch between degradative autophagy and secretory autophagy can be controlled (Zhang and Wang, 2018). GORASP2 is involved in the secretion of IL-1B, HMGB1 (Li et al., 2022) and TGFB1 (Nuchel et al., 2018). In response to glucose starvation, GORASP2 is de-O-GlcNAcylated (Zhang et al., 2018), and this serves to cater for the cell’s needs by inducing autophagosome-lysosome fusion and a rapid switching from extracellular release to cargo degradation and recycling. Perhaps some cargos such as IL1B are by default sent for degradation and only upon stimulation involving selective receptors such as TRIM16, is the IL1B sequestered into plasma membrane destined vesicles. Whether these two autophagy-dependent arms can be co-coordinated to run in parallel, or whether one dominates at the expense of the other, remains to be clarified in the coming years through further elucidation of upstream stimuli and molecular players that can guide the intracellular switch.

A number of loss-of-function studies have clarified the importance of the lipidation machinery in mediating SA and have helped to underline the pathological consequences of SA dysfunction, particularly in the area of cancer. SA of HMGB1 has been linked to several crucial roles in cancer progression both in the cancer cells themselves as well as in the tumor microenvironment. In different cell types, including glioblastoma cells or cancer associated fibroblasts (CAFs), HMGB1 secretion was dependent on the lipidation machinery components such as ATG5 (Ren et al., 2021; Li et al., 2022). Moreover, Ren et al. showed that ATG5 KO in CAFs decreased autophagy-dependent release of HMGB1 and the subsequent induction of epithelial-to-mesenchymal transition (EMT). In mouse mammary fibroblasts with ATG12 KO, IL6 secretion was inhibited, without affecting total levels, directly impacting tumors by reducing angiogenesis and tumor growth (Rudnick et al., 2021). SA plays a clear role in both the recruitment of CD4^+^, CD3^+^ and F4/80 + immune cells and the differentiation of tumor associated macrophages (TAMs) into an M1 anti-tumor phenotype (Li et al., 2022) as well as promoting the pro-inflammatory actions of CD4^+^ T-cells. LC3B+, but not LC3B- vesicles derived from B16F10 melanoma cells induced IL6 expression in CD4^+^ T-cells, again suggesting the direct involvement of ATG8 lipidation in secretion (Chen et al., 2019). Here HSP90A on the vesicle surface acts as a ligand to the TLR2 receptor on T-cells to induce the secretion of interleukins *via* NF-KB signaling. This was partly responsible for increased tumor growth and metastasis by suppression of anti-tumor immune signaling. Targeting autophagosome formation in B16F10 tumor cells resulted in reduced tumor growth in mice (Chen et al., 2019).

Recently TGFB1 has been shown to be released by SA, which has many known effects in cancer and the tumor microenvironment. TGFB1 secretion is abolished in fibroblasts and macrophages where key autophagy regulators such as ATG5 and ATG7 are ablated (Nuchel et al., 2018), hinting that targeting TGFB1 release by SA may be beneficial in cancer.

These studies demonstrate that targeting autophagy does not necessarily need to entirely focus on the cancer cells themselves. Altering autophagy within the microenvironment is enough to limit overall tumor progression. But the intricate interplay of different cell types requires careful targeting approaches.

Secretory autophagy is also required for the efficient secretion of cargo other than protein, including damaged mitochondria (Tan et al., 2022), ATP, DNA (Jeppesen et al., 2019) and several types of RNA (Leidal et al., 2020). Liedal et al. performed RNA-Seq on extracellular vesicles identifying hundreds of microRNAs (miRNAs) and small nucleolar RNAs (snoRNAs) among secreted cargo in a process that was ATG7 or ATG12-dependent, but independent of upstream autophagy regulators such as RB1-inducible coiled-coil protein 1 (FIP200), a key component of the ULK1 complex. For instance, they found that while snoRNAs made up 23% of secreted RNA in the control cells, this was reduced to just 6% in ATG7 KO cells (Leidal et al., 2020). This study provides an example of how specified types of secretory autophagy may tune intra- and extracellular pools of certain substrates such as RNA, a topic which is further discussed below.

## Autophagy, ATG8s and RNA homeostasis

The autophagic potential for altering RNA-metabolism has received increased attention in recent years, shedding light on both canonical and non-canonical mechanisms that lead to RNA-decay (Abildgaard et al., 2020). RNA is an understudied autophagy substrate, even though it was first shown to be degraded by lysosomes during amino acid starvation in rat livers many years ago (Lardeux et al., 1987; Heydrick et al., 1991). Later findings from *S. cerevisiae* have confirmed that RNA is degraded by autophagy during nitrogen starvation (Huang et al., 2015b) and that specific mRNAs can be selectively targeted by canonical autophagy (Makino et al., 2021). In the latter study, vacuoles were purified by ultracentrifugal flotation from yeast strains lacking the vacuolar RNase rny1 and treated with rapamycin. Sequencing of the vacuolar mRNA content showed an enrichment of mRNAs coding for ribosomal proteins and proteins involved in amino acid biogenesis. This selectivity was independent of basal mRNA abundance in the cells. Interestingly, vacuolar-enriched mRNAs maintained an association to ribosomes after rapamycin treatment that was not observed among vacuolar-depleted RNAs, suggesting a potential inter-regulation between translation and vacuolar RNA sequestration (Makino et al., 2021). In *Arabidopsis Thaliana* it was similarly shown that certain RNAs are recruited for autophagic degradation. Specifically, the authors found that chloroplast encoded rRNA and miRNA were significantly enriched in vacuoles from WT plants relative to ATG5 knockout plants upon starvation-induced autophagy (Hickl et al., 2021). Interestingly, the majority of the vacuolar RNAs were pre-tRNA, for which vacuolar sequestering was independent of ATG5, suggesting an alternative unknown mechanism of RNA-delivery for certain RNA species. These findings from both yeast and plants suggest that autophagy may contribute to stress-induced transcriptome and translatome rewiring through selective decay of mRNAs and/or non-coding RNA decay, a topic recently discussed in further detail elsewhere (Abildgaard et al., 2020; Kumaran and Michaeli, 2021).

Although the ability of autophagy to degrade RNA in human cells is not well-studied, it is interesting to note that key autophagy proteins including p62 and LC3B are RNA binding proteins (Zhou et al., 1997; Horos et al., 2019; Hwang et al., 2022). It was recently shown that LC3B can mediate RNA degradation by a lysosome-independent pathway, through interaction with the CCR4-NOT adenylase complex (Hwang et al., 2022). In this study, LC3B and additional proteins of the lipidation machinery (ATG5, ATG12 and ATG16L1) were found to colocalize and interact with CNOT1 and CNOT7 from the CCR4-NOT complex upon induction of autophagy to shorten the length of poly-A on the targeted mRNAs, leading to rapid mRNA-decay. Loading of LC3B onto mRNA was further shown to be dependent on the 3′UTR AAUAAA consensus motif found close to the polyadenylation signal (Hwang et al., 2022). In contrast, mRNAs were shown to be selectively degraded in a 5′UTR-dependent manner by canonical autophagy in yeast (Makino et al., 2021). CCR4-NOT dependent degradation was further shown to target *PRMT1* mRNA for degradation, which in turn lead to an increased autophagy response (Hwang et al., 2022). These studies suggest not only that RNA serves as a specific autophagy substrate, but that additional non-canonical functions of ATG8 proteins, through previously unknown complex interactions, provide a means for specific RNA degradation during cellular stress, possibly as a way to alter global cellular RNA homeostasis as an adaptive response to environmental threats.

## Concluding remarks

The topics discussed in this review leave us with several open questions. What are the decisive cues that coordinate the multiple autophagy-dependent and independent functions of ATG8s? How do ATG8s know where to go and when? To what extent are individual ATG8 family member roles functionally pre-specified and in which context can they be redundant? Adding a layer of complexity to this, our knowledge of ATG8 post-translational modifications is beginning to reveal how these may impact structural properties of ATG8s, which in turn can influence aspects related to subcellular localization and key interaction partners. In line with these considerations, it will be intriguing to uncover how ATG8 family members and their individual interactomes differ from one another at subcellular locations or on distinct membranes, to clarify some of these poorly understood functional discrepancies. Moreover, how the decisions are made for secretory versus degradative outcomes and the interplay between these two fates remain unclear. Further investigation of the questions and concepts put forward here, will undoubtedly open new and exciting lines of research in the autophagy field, and will importantly also elucidate how the autophagy machinery can be utilized for other purposes. We are likely only beginning to uncover the widespread physiological implications of these pathway alternatives in health and disease.
